# A machine learning model for diabetic retinopathy risk stratification using routine blood and urine parameters: insights into kidney-eye crosstalk

**DOI:** 10.3389/fendo.2026.1843412

**Published:** 2026-06-17

**Authors:** Yong Wang, Ling Yao, Tianpeng Chen, Qianyu Zhang, Xin Cao, Jiayi Gu, Sijie Bao, Xiaojuan Chen, Cheng Cao

**Affiliations:** 1 Department of Ophthalmology, Affiliated Nantong Clinical College of Nantong University, Nantong First People’s Hospital, Nantong, Jiangsu, China; 2 Department of Ophthalmology, Nantong First People’s Hospital, Nantong, China; 3 Medical Research Center, Nantong First People’s Hospital, Nantong, China

**Keywords:** clinical decision support, diabetic retinopathy, interpretable machine learning, risk prediction, routine laboratory biomarkers

## Abstract

**Objective:**

This study aimed to develop and externally validate an interpretable machine learning (ML) model for diabetic retinopathy (DR) risk stratification using routine clinical biomarkers, and to explore potential probabilistic dependencies and interactive pathways between clinical biomarkers and DR pathogenesis through Bayesian network modeling.

**Methods:**

We integrated clinical data from the National Health and Nutrition Examination Survey (NHANES) with an independent hospital cohort (Nantong First People’s Hospital). A multi-stage feature selection pipeline (Boruta algorithm and LASSO regression) was utilized to identify core predictors. Eight ML algorithms were benchmarked. To transcend conventional “black-box” predictions, we coupled SHAP (SHapley Additive exPlanations) for personalized interpretability with a Bayesian Network Directed Acyclic Graph (DAG) to map the probabilistic dependency structure among the selected systemic biomarkers.

**Results:**

The LightGBM algorithm outperformed other classifiers, yielding a robust external validation AUC of 0.841 (95% CI: 0.809-0.862). Fourteen key routine predictors were identified, spanning glycemic control, renal function, and lipid metabolism. Crucially, probabilistic dependency structure via the Bayesian Network revealed a hierarchical pathogenetic topology: rather than parallel associations, latent renal impairment markers (urine protein, BUN, and urine creatinine) and chronic glycemic toxicity (HbA1c) emerged as direct upstream dependency drivers of DR. This structural evidence suggests a probabilistic dependency consistent with the ‘kidney-eye crosstalk’ hypothesis.

**Conclusion:**

We successfully deployed a high-performing, non-invasive LightGBM model for early DR screening. By integrating predictive ML with probabilistic dependency structure, this framework not only delivers an accessible, web-based clinical decision support system (CDSS) for resource-constrained settings but also provides preliminary insights into the potential systemic microvascular interplay driving diabetic retinopathy.

## 1 Introduction

Diabetic retinopathy, the most prevalent microvascular complication of diabetes mellitus, remains the leading cause of vision loss among working-age adults worldwide. With a global prevalence exceeding 103 million cases, DR represents a major preventable cause of blindness in this population and imposes substantial public health burdens (1). The global diabetes epidemic is projected to affect approximately 700 million individuals by 2045, with the prevalence of DR expected to rise to around 160 million, presenting an unprecedented challenge to healthcare systems internationally (1, 2). The pathogenesis of DR involves a complex, multifactorial process mediated through multiple interrelated pathways. In addition to established risk factors such as duration of diabetes, glycemic control (as reflected by HbA1c levels), and hypertension, emerging evidence has increasingly linked novel biomarkers to the development and progression of DR (3). Chronic hyperglycemia-induced oxidative stress and inflammatory responses are recognized as central mechanisms underlying DR onset and advancement (4). Despite significant therapeutic advances—including anti-VEGF therapy, laser photocoagulation, and vitreoretinal surgery—current management strategies remain largely reactive, focusing on late-stage complications rather than targeting early pathophysiological changes (5, 6). This limitation results in suboptimal clinical outcomes and insufficient preventive interventions, highlighting the critical need for early detection tools based on accessible biomarkers and validated predictive models.

Early detection and timely intervention can reduce the risk of severe vision loss due to DR by up to 90% (7). Current screening methods primarily rely on fundus examination and fundus photography. Fundus examination, performed by ophthalmologists or optometrists, requires specialized instruments such as binocular indirect ophthalmoscopes or slit lamps, along with advanced clinical expertise. Fundus photography, typically conducted by trained personnel, depends on costly equipment including optical coherence tomography (OCT) devices and digital fundus cameras (8). Although the American Diabetes Association (ADA) recommends annual eye examinations as the gold standard for DR screening, adherence remains low, with fewer than 60% of diabetic patients complying with these guidelines. This gap is primarily attributed to the high cost and limited accessibility of ophthalmic services, particularly in underserved and rural areas (8, 9).

In recent years, artificial intelligence (AI) and machine learning (ML) technologies have demonstrated transformative potential in the prediction and diagnosis of DR. Deep learning models have achieved remarkable performance in automated DR screening and staging (10). However, most of these high-performing deep learning systems have been developed for image-based tasks, particularly fundus photography or OCT analysis, whereas their comparative utility in lower-dimensional structured biomarker datasets remains less well established. The integration of laboratory data with machine learning has been widely explored. For example, Yang et al. (11) developed a nomogram incorporating predictors such as diabetes duration, diabetic neuropathy, diabetic foot, diabetic nephropathy, hyperlipidemia, and hypoglycemic medication use. Feng He et al. (12) identified diabetes duration, insulin use, age, and circulating tyrosine as significant factors associated with both diabetic kidney disease (DKD) and DR. Moreover, the combination of ML with biomedical big data has enabled the discovery of novel biomarkers and improved diagnostic accuracy beyond traditional risk factors. Varun Gulshan et al. (13)developed a deep learning algorithm capable of detecting DR from retinal fundus photographs. However, despite these advancements, existing predictive models exhibit several limitations. Most rely heavily on retinal imaging data, which hinders their scalability in resource-limited settings. Many studies fail to fully leverage routinely available clinical and laboratory parameters for model development, limiting applicability in primary care environments. Furthermore, a majority of models lack rigorous external validation, raising concerns about their generalizability and real-world utility. Notably, conventional models often omit biomarkers reflecting emerging pathogenic mechanisms—such as those related to oxidative stress and insulin resistance—thereby constraining predictive performance and clinical relevance.

Recent advances in medical artificial intelligence have demonstrated strong performance in a wide range of diagnostic tasks, particularly in imaging-based disease classification and detection. For example, deep learning frameworks have shown promising results in brain tumor classification, breast cancer detection, and other disease-specific recognition settings, while increasingly incorporating explainability and robustness to facilitate clinical translation (14–17).

Moreover, studies have identified multiple differentially expressed proteins in blood (18) vitreous humor (19), retina (20), and tears (21) of DR patients and animal models, suggesting potential targets for early diagnosis and therapeutic intervention. Concurrently, research utilizing large-scale public databases such as NHANES has revealed associations between DR risk and various systemic factors, including sleep quality (22), blood cell differentials (23), and dietary patterns (24).

To address these gaps, this study aimed to develop a robust, interpretable, and clinically deployable risk prediction model for DR. Using data from the nationally representative National Health and Nutrition Examination Survey (NHANES), we systematically incorporated routine physical and laboratory measurements and extended the feature set to include recently reported composite indicators associated with DR. These encompassed domains such as obesity (e.g., Weight-Waist Index (WWI); LDL-to-HDL ratio (LHR); Triglyceride-Glucose Index (TyG)), oxidative stress (e.g., Aggregate Index of Systemic Inflammation (AISI); Systemic Immune-Inflammation Index (SII); Neutrophil-to-Lymphocyte Ratio (NLR); HbA1c-to-HDL Ratio(HHR); Platelet-to-Lymphocyte Ratio (PLR); Monocyte-to-Lymphocyte Ratio (MLR); Neutrophil-to-HDL Cholesterol Ratio (NHR); TyG-BMI; TyG-IR; A Body Shape Index (ABSI); TyG-ABSI), and insulin resistance (e.g., METS-IR; GLM7).

From a methodological perspective, this study leverages a rigorous, multi-stage feature selection pipeline—integrating the Boruta all-relevant algorithm with LASSO regression and multivariable logistic regression, to distill 14 core predictive variables from a high-dimensional clinical pool. These predictors encompass critical dimensions of metabolic health, including glycemic control (HbA1c, FBG, insulin), renal function (PRO, UCREA, BUN, creatinine, albumin), hemodynamics (DBP), electrolytes (sodium, potassium), and metabolic/oxidative stress markers (LHR, LDH, total bilirubin). Subsequently, eight machine learning architectures ranging from traditional Logistic Regression to advanced ensemble methods like XGBoost and LightGBM were systematically benchmarked. Among these, the LightGBM model demonstrated superior discriminative performance, achieving a robust area under the curve (AUC) of 0.849 in the internal cohort. Crucially, independent external validation at the First People’s Hospital of Nantong confirmed the model’s exceptional generalizability, yielding a stable AUC of 0.841.

A distinctive innovation of this research lies in its dual-layer interpretability framework. Beyond conventional predictive modeling, we coupled SHAP-based global explanations with Bayesian Network probabilistic dependency structure to untangle the complex pathogenetic interdependencies between these markers. Our findings highlight a synergistic predictive value between traditional laboratory metrics and novel composite indicators, such as the LDL-to-HDL ratio (LHR). Specifically, the probabilistic topology suggested that systemic lipid perturbations and hemodynamic shifts are structurally mediated through latent renal impairment (e.g., proteinuria) to drive retinal microangiopathy. This observation aligns with recent multi-omics and Mendelian randomization studies, which underscore the integrated role of the “kidney-eye crosstalk” and lipid dysregulation in the pathogenesis of diabetic microvascular complications.

The theoretical and practical contributions of this study are threefold. First, the proposed LightGBM framework enables precise DR risk stratification relying exclusively on routinely accessible clinical parameters, thereby eliminating the prerequisite for high-cost fundus imaging and facilitating large-scale screening in resource-constrained primary care settings. Second, the minimal performance decay between internal and external cohorts validates the model’s structural resilience and cross-population robustness. Third, by bridging the gap between algorithmic “black-box” predictions and biological causality through Bayesian modeling, this study offers supplementary clinical insights into the multifactorial etiology of DR. Collectively, this work not only delivers a scalable, cost-effective clinical decision support tool but also proposes a data-driven framework for future pathophysiological investigations into diabetic complications.

## 2 Methods

### 2.1 Study population and design

**Figure 1:**
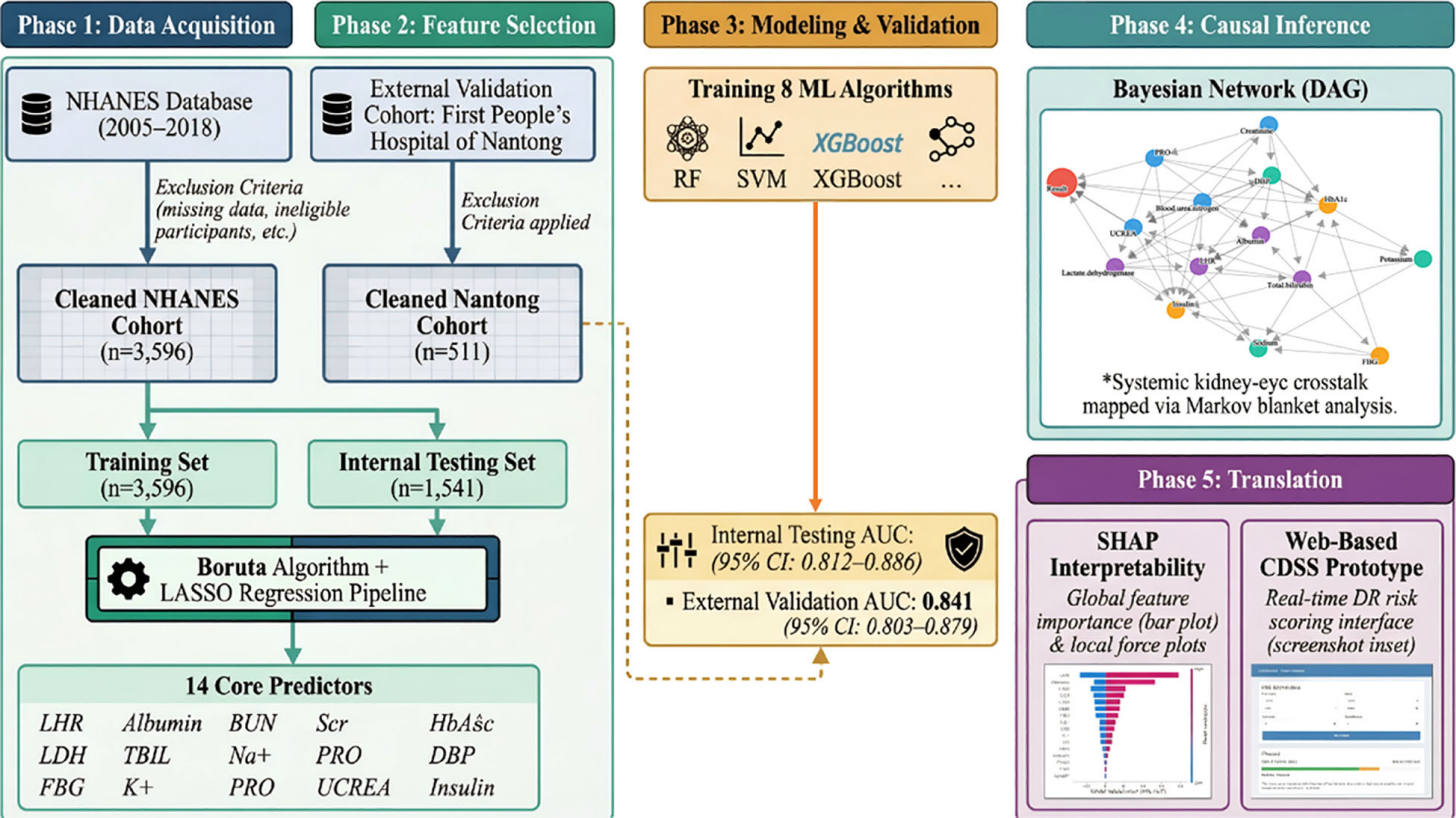
Flow chart of participants selection. NHANES, National Health and Nutrition Examination Survey.

This study constitutes a retrospective, multicenter investigation focused on model development and validation. The study cohorts were derived from two independent datasets. The model development cohort utilized publicly available data from the NHANES collected between 2005 and 2018. NHANES employs a complex, stratified, multi-stage probability sampling design to assess the health and nutritional status of non-institutionalized adults and children in the United States, rendering its data nationally representative (25). The external validation cohort consisted of consecutive medical records of diabetic patients admitted to the Department of Ophthalmology at the First People’s Hospital of Nantong, China, from July 2025 to December 2025. The study protocol received approval from the Institutional Review Board of the Nantong First People’s Hospital (Ethics Approval No.: 2026-KT015-02), and informed consent was obtained from all participants (**Figure 1**).

### 2.2 Data collection and clinical definitions

Adult participants with diabetes mellitus were identified from NHANES based on the available diabetes-related information in the dataset. Because NHANES does not provide a uniformly validated classification of diabetes subtype for all participants, type 1 and type 2 diabetes were not strictly separated in this study; however, given the adult study population, the cohort is expected to predominantly represent individuals with type 2 diabetes.

Definition of DR: In the NHANES dataset, diabetic retinopathy status was defined using the questionnaire variable DIQ080 (“Diabetes affected eyes/had retinopathy”). Participants who answered “Yes” were classified as having DR. In the external validation cohort from Nantong Hospital, DR diagnosis was established through dilated fundus examination and independently confirmed by two senior ophthalmologists. Fundus fluorescein angiography (FFA) and optical coherence tomography (OCT) were performed only when clinically indicated, such as in cases requiring further evaluation of retinal lesions or macular involvement, and were not mandatory for DR diagnosis in all patients.

Predictors and Calculation Formulas: This study incorporated the following four categories of predictors, derived from demographic, clinical, and laboratory data:(1) Conventional Demographic and Laboratory Indicators: Age, sex, duration of diabetes, systolic and diastolic blood pressure, serum creatinine, serum potassium, FBG, blood urea nitrogen, total bilirubin, lactate dehydrogenase, urine protein, and glycated hemoglobin. (2) Obesity-Related Indices: Weight-Waist Index (WWI) = Waist circumference (cm)/√Weight (kg); Triglyceride-Glucose Index (TyG) = ln(Fasting triglycerides (mg/dL) × Fasting glucose (mg/dL)/2); (3) Oxidative Stress and Systemic Inflammation Indices (calculated from complete blood count parameters): Aggregate Index of Systemic Inflammation (AISI) = (Neutrophils × Platelets × Monocytes)/Lymphocytes; Systemic Immune-Inflammation Index (SII) = (Platelets × Neutrophils)/Lymphocytes;Platelet-to-Neutrophil Ratio (PNR) = Platelets/Neutrophils;Neutrophil-to-Lymphocyte Ratio (NLR) = Neutrophils/Lymphocytes; Monocyte-to-Lymphocyte Ratio (MLR) = Monocytes/Lymphocytes; Neutrophil-to-HDL Cholesterol Ratio (NHR) = Neutrophils/HDL-C; LDL-to-HDL Ratio (LHR) = LDL/HDL; HbA1c-to- HDL Ratio (HHR) = HbA1c/HDL; (4) Insulin Resistance and Derived Composite Indices: Metabolic Syndrome–Derived Insulin Resistance Index (METS-IR) = ln((2 × FPG (mg/dL)) + TG (mg/dL)) × BMI (kg/m^2^)/ln(HDL-C (mg/dL)); Glucose-Lipid Metabolism Index 7 (GLM7) = log_10_(Age (years) × BMI (kg/m^2^) × FBG (mg/dL) × Insulin (pmol/L) × TG (mmol/L) × LDL-C (mmol/L)/HDL-C (mmol/L)).

Additional Derived Indices: TyG-BMI = TyG index × BMI; TyG-IR is equivalent to the TyG index; A Body Shape Index (ABSI) = Waist circumference/(BMI^(2/3) × Height^(1/2)); TyG-ABSI = TyG index × ABSI.

### 2.3 Inclusion and exclusion criteria

For both cohorts, the inclusion criteria were: (1) age ≥ 18 years; and (2) a confirmed diagnosis of diabetes mellitus according to the criteria established by the American Diabetes Association or the World Health Organization. The exclusion criteria included: (1) missing data on key variables, such as DR status, fasting blood glucose, or complete blood count parameters; (2) presence of severe systemic conditions that could significantly affect retinal or inflammatory biomarkers, including active malignancy, end-stage renal disease, or autoimmune disorders; and (3) history of ocular trauma or non-diabetic retinal diseases.

### 2.4 Feature selection and model development

Diabetes duration was initially considered as a candidate predictor; however, it was excluded from the final analysis because it was not consistently available in the external validation cohort, and incomplete self-reported duration data may introduce recall bias. Prior to modeling, continuous variables were preprocessed to improve comparability and model stability. Continuous variables with markedly right-skewed distributions were log-transformed to reduce skewness before analysis. Subsequently, these transformed variables were standardized together with the remaining continuous predictors for machine learning modeling.

The modeling workflow was executed through a systematic and iterative process. To address class imbalance, SMOTE oversampling was applied only to the training set during model development. No oversampling or synthetic sample generation was performed in the internal testing set or the external validation set, thereby avoiding information leakage and preserving unbiased performance evaluation. Initially, all continuous variables were standardized to ensure comparability across different scales, followed by a preliminary screening using differential analysis and initial logistic regression to identify variables with potential predictive value. To address the risk of multicollinearity and redundant information, a pairwise correlation analysis was performed; in instances of high feature redundancy, the variable demonstrating a weaker association with the outcome was systematically excluded, ensuring that only the most informative markers were retained.

To improve feature selection robustness, we adopted a multistage strategy integrating Boruta, LASSO, and multivariable logistic regression. Boruta was used to identify all-relevant candidate variables, LASSO to reduce dimensionality and handle collinearity, and multivariable logistic regression to confirm independent associations within a conventional clinical-statistical framework. Final predictors were defined as the intersection of these methods to enhance selection stability and interpretability. A threshold of P < 0.1 was used in multivariable logistic regression to avoid prematurely excluding potentially meaningful predictors in the presence of correlated biomarkers and possible mediation effects. Following this initial filtration, a rigorous multi-stage feature selection pipeline was implemented to refine the predictive variables. The Boruta algorithm, a random forest-based wrapper method, was first employed to capture all relevant features from the extensive pool. These candidates were then subjected to LASSO regression, where the optimal penalty parameter (λ) was determined via 10-fold cross-validation to further shrink non-essential coefficients to zero. Finally, the variables retained by LASSO were validated through univariate logistic regression, with only those maintaining a significance level of P<0.1 forming the definitive set of 14 core predictors, including the LDL-to-HDL ratio (LHR) and other metabolic indicators.

To assess the robustness of the selected predictors beyond the single 70/30 train-test split, we performed a resampling-based stability analysis using the NHANES dataset. Repeated bootstrap resampling was conducted, and in each resample the 14 candidate core predictors were re-evaluated using LASSO logistic regression and multivariable logistic regression. Selection frequencies were then calculated for each predictor across all resampling iterations. Predictors with a selection frequency of at least 70% were considered stable. The selection frequencies were as follow: BUN (100%), PRO (98%), HbA1c (97%), LDH (92%), Creatinine (90%), FBG (89%), UCREA (86%), Albumin (81%), Insulin (79%), Sodium (74%), DBP (71%), Potassium (69%), LHR (65%), and Bilirubin_Total (54%). These results indicate that the major predictors identified in our study remained reproducible across repeated resampling and were not merely artifacts of the original single random split. Eight distinct machine learning classifiers—Logistic Regression, SVM, KNN, Decision Tree, Random Forest, Neural Network, XGBoost, and LightGBM—were trained using the primary dataset. To maximize the discriminative capacity of each model, hyperparameters such as learning rate, tree depth, and regularization strength were meticulously optimized using a grid search strategy combined with 10-fold cross-validation. This approach ensured model stability and minimized the risk of overfitting during the training process.

In the final evaluation stage, the performance of the optimized models was comprehensively assessed using the internal testing set and an independent external validation cohort. The primary metric for discriminative power was the Area Under the Receiver Operating Characteristic Curve (AUC), supplemented by sensitivity, specificity, accuracy, and the F1-score. Furthermore, Decision Curve Analysis (DCA) was utilized to determine the net clinical benefit of the models across varying threshold probabilities, thereby validating their practical utility for clinical decision support in real-world settings (23).

### 2.5 Model explanation

To enhance the interpretability of machine learning models, the SHapley Additive exPlanations (SHAP) method employs a two-tier analytical framework designed to address the “black box” nature of predictive algorithms and improve transparency in model predictions (26). This emphasis on explainability is increasingly aligned with broader developments in medical AI, where transparent and clinically interpretable model behavior is regarded as essential for real-world adoption (27). At the global level, SHAP computes the average contribution of each feature to identify key predictors of DR; at the individual level, it reveals the rationale behind specific predictions by analyzing the influence of input variables on case-by-case outcomes, thus providing patient-specific explanations. This dual-level approach enhances understanding of the model’s decision-making process and strengthens its potential for reliable clinical implementation (28).

### 2.6 External validation

To rigorously evaluate the model’s generalization capability, the finalized LightGBM model—including its hyperparameter configuration and selected features—was directly applied to an external validation cohort from Nantong First People’s Hospital without any retraining or recalibration. Performance metrics, including the AUC, were computed in this independent population to assess the model’s clinical generalizability.

### 2.7 Bayesian network construction and probabilistic dependency analysis

To transcend the purely correlative nature of predictive machine learning and map the mechanistic pathways driving diabetic retinopathy (DR), we constructed a Bayesian Network (BN) Directed Acyclic Graph (DAG). The BN analysis was exclusively conducted on the 14 optimal core predictors identified by our feature selection pipeline, alongside the clinical outcome (DR status), to ensure topological stability and interpretability.

Structure learning was performed using the score-based Hill-Climbing (HC) algorithm. To guarantee the structural robustness of the learned directed dependency structure and mitigate sample-specific biases, we employed a bootstrap resampling approach with 100 iterations. An averaged consensus network was subsequently derived, retaining only those directed arcs (edges) that demonstrated high statistical stability across the bootstrap samples.

A critical methodological strength of our directed dependency structure was the integration of data-driven learning with clinical and algorithmic priors. The Bayesian network was learned using a hybrid strategy that combined data-driven structure discovery with limited clinically informed prior constraints. Specifically, PRO, HbA1c, BUN, and LHR were specified as direct parent nodes of DR based on their strong clinical plausibility and consistent importance in the preceding statistical analyses, in order to avoid clinically implausible edge orientations in a purely unconstrained observational network. First, a structural “blacklist” was strictly enforced to prohibit biologically implausible directed edges originating from the clinical outcome (DR) to any baseline systemic biomarker. Second, to overcome the well-documented issue of collinearity masking among highly correlated physiological indices (e.g., renal and glycemic markers), we established a targeted “whitelist”. Informed by our SHAP value rankings and established pathophysiological mechanisms, the most dominant predictive drivers (specifically PRO, HbA1c, blood urea nitrogen, and LHR) were structurally constrained as direct upstream parent nodes to the DR outcome.

This hybrid approach ensures that the resulting probabilistic dependency architecture is both statistically rigorous and clinically sound. All BN computations were executed using the bnlearn package (version 4.9.3) in R, and the final DAG was modularly visualized utilizing the igraph and ggraph packages, with nodes categorized by physiological systems to facilitate pathophysiological interpretation.

### 2.8 Webpage deployment tool

To facilitate clinical implementation, the finalized model was deployed via a web-based clinical decision support tool (29). The system provides (1): DR probability estimates (0%-100%) within 24 h admission using clinical and biomarker inputs, and (2) patient-specific prediction explanations for risk. For deployment in the web-based calculator, user-entered values were processed using the same preprocessing logic as in model development. Specifically, out-of-range inputs were first truncated to the nearest allowable boundary, and missing values were imputed according to the predefined preprocessing rules before standardization and model inference. This sequence was adopted to ensure consistency with the model training domain, avoid unstable extrapolation from implausible raw inputs, and preserve numerical stability of the generated risk estimates.

### 2.9 Statistical analyses and data preprocessing

All statistical analyses and machine learning computations were conducted using R software (version 4.2.2). Baseline demographic and clinical characteristics were summarized according to data type: continuous variables are expressed as means ± standard deviations (SD), while categorical variables are presented as frequencies and percentages (n [%]). For group comparisons, differences between the non-DR and DR cohorts were evaluated using the independent Student’s t-test or Mann-Whitney U test for continuous data, and the Chi-square test or Fisher’s exact test for categorical data, as appropriate based on normality. A two-sided P-value < 0.05 was considered statistically significant.

Prior to model construction, a rigorous data preprocessing pipeline was executed to ensure dataset integrity and optimize algorithmic performance. To address missing values without compromising the statistical power of the cohort, we applied a non-parametric imputation method utilizing the missForest package. This Random Forest-based algorithm was specifically selected for its robustness in handling complex, non-linear interactions among diverse clinical variables.

Following data imputation, we addressed the inherent class imbalance between the disease and control groups. The Synthetic Minority Over-sampling Technique (SMOTE) was deployed to rebalance the training dataset. By generating synthetic instances of the minority class based on feature space similarities—rather than relying on simple observational duplication—this approach effectively mitigated the risk of the algorithms developing a predictive bias toward the majority class, thereby reducing overfitting. Furthermore, to eliminate potential biases introduced by the disparate measurement scales of routine laboratory biomarkers, all continuous features underwent Z-score standardization, transforming them to a uniform distribution with a mean of 0 and a standard deviation of 1. This standardized, balanced, and complete dataset subsequently served as the foundation for the multi-stage feature selection and model training phases.

## 3 Results

### 3.1 Demographic characteristics of participants

A total of 5,137 diabetic patients were included in this study, comprising 4,042 individuals in the No-DR group and 1,095 in the With-DR group. Initially, demographic analysis revealed that patients with DR were significantly older (P = 0.023) and exhibited a higher prevalence of hypertension (P = 0.003) compared to the No-DR cohort. Socioeconomic factors, including marital status and lower annual household income, also showed significant associations with DR presence (all P<0.05).

Notably, several of the 14 core predictors identified for our final model demonstrated marked baseline differences. Regarding glycemic control and insulin resistance, the DR group presented significantly elevated levels of FBG, HbA1c, and insulin-related indices (e.g., METS-IR) compared to the No-DR group (all P<0.05). Similarly, markers of renal dysfunction and protein excretion—critical components of our predictive model—were significantly altered in the DR group, characterized by higher BUN, serum creatinine, and UCREA, alongside a higher frequency of proteinuria (PRO) (all P<0.001).

Furthermore, systemic metabolic and electrolyte imbalances were evident. The DR group exhibited significantly lower levels of serum albumin, sodium, and total calcium, while displaying higher levels of uric acid and alkaline phosphatase (all P<0.05). Although individual lipid parameters such as LDL-C and HDL-C did not reach statistical significance independently (P>0.05), the LHR—a key composite indicator in our model—reflected a complex lipid perturbation associated with DR. Additionally, blood pressure profiles differed significantly, with the DR group showing elevated systolic blood pressure but lower DBP (P = 0.002).

Finally, markers of systemic inflammation and immune status, including the NLR, MLR, and SII, were significantly higher in the DR group, reinforcing the role of chronic inflammation in the pathogenesis of retinopathy. In contrast, no significant intergroup differences were observed in BMI, waist circumference, smoking status, or alcohol consumption (P>0.05). Collectively, these baseline disparities across glycemic, renal, inflammatory, and hemodynamic dimensions provide a robust physiological rationale for the 14 variables utilized in our LightGBM-based predictive framework. (**Table 1**).

**Table 1:** Baseline characteristics of the population in two cohorts.

Variables	Total (n = 5137)	No-DR (n = 4042)	With-DR (n = 1095)	P
Age (years), Mean ± SD	61.59 ± 13.85	61.37 ± 14.12	62.38 ± 12.76	0.023
White blood cell count (1000 cells/uL), Mean ± SD	7.58 ± 2.27	7.59 ± 2.30	7.54 ± 2.15	0.541
Segmented neutrophils num (1000 cell/uL), Mean ± SD	4.55 ± 1.66	4.54 ± 1.66	4.58 ± 1.69	0.450
Lymphocyte percent (%), Mean ± SD	2.19 ± 1.10	2.21 ± 1.16	2.11 ± 0.85	0.013
Monocyte percent (%), Mean ± SD	0.58 ± 0.20	0.58 ± 0.20	0.57 ± 0.19	0.411
Platelet count (1000 cells/uL), Mean ± SD	240.53 ± 69.73	240.82 ± 69.46	239.44 ± 70.74	0.561
Weight (kg), Mean ± SD	89.54 ± 23.43	89.47 ± 23.10	89.80 ± 24.61	0.697
Waist (cm), Mean ± SD	109.33 ± 15.83	109.20 ± 15.62	109.81 ± 16.59	0.258
Height (cm), Mean ± SD	166.15 ± 10.08	166.26 ± 10.13	165.77 ± 9.91	0.157
BMI(kg/m2), Mean ± SD	32.32 ± 7.44	32.26 ± 7.33	32.54 ± 7.85	0.272
WWI, Mean ± SD	11.64 ± 0.71	11.63 ± 0.71	11.68 ± 0.72	0.032
HDL (mg/dL), Mean ± SD	48.47 ± 13.76	48.44 ± 13.70	48.59 ± 14.00	0.751
Triglyceride (mg/dL), Mean ± SD	163.54 ± 113.99	162.48 ± 114.51	167.41 ± 112.01	0.204
LDL (mg/dL), Mean ± SD	102.00 ± 25.38	102.10 ± 25.26	101.62 ± 25.85	0.577
AISI, Mean ± SD	332.90 ± 285.35	329.44 ± 283.21	345.66 ± 292.91	0.095
SII, Mean ± SD	562.42 ± 371.24	554.86 ± 359.81	590.31 ± 409.65	0.009
PNR, Mean ± SD	58.78 ± 26.23	59.13 ± 26.91	57.48 ± 23.51	0.065
MLR, Mean ± SD	0.29 ± 0.14	0.29 ± 0.14	0.30 ± 0.15	0.006
NLR, Mean ± SD	2.36 ± 1.41	2.32 ± 1.36	2.49 ± 1.58	<0.001
NHR, Mean ± SD	0.10 ± 0.05	0.10 ± 0.05	0.10 ± 0.05	0.483
SIRI, Mean ± SD	1.38 ± 1.03	1.36 ± 1.03	1.44 ± 1.03	0.032
LHR, Mean ± SD	2.25 ± 0.80	2.26 ± 0.80	2.65 ± 0.82	0.089
TyG, Mean ± SD	9.30 ± 0.67	9.27 ± 0.65	9.38 ± 0.71	<0.001
TyG-BMI, Mean ± SD	300.89 ± 74.37	299.54 ± 72.74	305.83 ± 79.96	0.019
METS-IR, Mean ± SD	51.65 ± 13.75	51.42 ± 13.44	52.51 ± 14.83	0.028
ABSI, Mean ± SD	0.06 ± 0.01	0.06 ± 0.01	0.06 ± 0.01	0.822
TyG-ABSI, Mean ± SD	0.58 ± 0.08	0.58 ± 0.08	0.58 ± 0.08	0.006
Albumin(g/dL), Mean ± SD	4.11 ± 0.35	4.12 ± 0.34	4.05 ± 0.37	<0.001
Alkaline phosphatase(IU/L), Mean ± SD	77.13 ± 32.44	76.46 ± 33.36	79.61 ± 28.69	0.004
Blood urea nitrogen(mg/dL), Mean ± SD	16.94 ± 8.55	16.25 ± 7.46	19.49 ± 11.36	<0.001
Total calcium (mg/dL), Mean ± SD	9.42 ± 0.39	9.43 ± 0.38	9.38 ± 0.43	<0.001
Creatinine(mg/dL), Mean ± SD	1.05 ± 0.77	0.99 ± 0.63	1.24 ± 1.12	<0.001
Glutamyl transferase (U/L), Mean ± SD	35.14 ± 47.66	35.05 ± 49.98	35.46 ± 37.88	0.803
Glucose (mg/dL), Mean ± SD	153.12 ± 72.11	149.30 ± 67.69	167.18 ± 85.06	<0.001
Iron (ug/dL), Mean ± SD	77.61 ± 29.86	77.99 ± 29.72	76.19 ± 30.32	0.077
Lactate dehydrogenase (U/L), Mean ± SD	138.23 ± 36.58	136.55 ± 31.06	144.44 ± 51.68	<0.001
Phosphorus (mg/dL), Mean ± SD	3.73 ± 0.58	3.72 ± 0.57	3.76 ± 0.62	0.017
Total bilirubin (mg/dL), Mean ± SD	0.62 ± 0.28	0.62 ± 0.29	0.60 ± 0.25	0.009
Uric acid (mg/dL), Mean ± SD	5.74 ± 1.53	5.71 ± 1.49	5.84 ± 1.67	0.019
Sodium (mmol/L), Mean ± SD	139.01 ± 2.75	139.09 ± 2.69	138.70 ± 2.92	<0.001
Potassium (mmol/L), Mean ± SD	4.10 ± 0.39	4.09 ± 0.38	4.15 ± 0.42	<0.001
Fluoride(umol/L), Mean ± SD	102.39 ± 3.47	102.46 ± 3.41	102.14 ± 3.69	0.009
Globulin (g/dL), Mean ± SD	3.04 ± 0.48	3.03 ± 0.48	3.06 ± 0.49	0.026
PRO(mg/L), Mean ± SD	17.90 (7.00, 63.00)	16.00 (6.60, 51.05)	29.60 (9.00, 134.30)	<0.001
UCREA(mg/dL), Mean ± SD	115.12 ± 69.09	117.14 ± 69.50	107.70 ± 67.09	<0.001
HbA1c (%), Mean ± SD	7.41 ± 1.73	7.32 ± 1.71	7.76 ± 1.78	<0.001
HHR, Mean ± SD	0.17 ± 0.06	0.16 ± 0.06	0.17 ± 0.07	<0.001
SBP(mmHg), Mean ± SD	131.66 ± 19.49	131.20 ± 18.96	133.34 ± 21.26	0.003
DBP(mmHg), Mean ± SD	68.17 ± 13.59	68.48 ± 13.36	67.00 ± 14.36	0.002
Blood cadmium (nmol/L), Mean ± SD	0.48 ± 0.44	0.47 ± 0.45	0.49 ± 0.43	0.264
Blood lead (μg/dL), Mean ± SD	1.57 ± 1.32	1.55 ± 1.32	1.64 ± 1.32	0.038
Blood mercury, total (μg/dL), Mean ± SD	1.46 ± 2.26	1.46 ± 2.28	1.45 ± 2.16	0.927
Total Cholesterol (mg/dL), Mean ± SD	181.03 ± 44.27	181.13 ± 43.86	180.64 ± 45.77	0.741
Fasting Glucose (mg/dL), Mean ± SD	160.01 ± 64.55	156.51 ± 61.47	172.93 ± 73.43	<0.001
Insulin (μU/mL), Mean ± SD	131.36 ± 151.11	126.80 ± 142.06	148.19 ± 179.73	<0.001
CHG, Mean ± SD	5.64 ± 0.54	5.63 ± 0.53	5.70 ± 0.57	<0.001
GLM7, Mean ± SD	9.94 ± 0.60	9.92 ± 0.59	10.01 ± 0.64	<0.001
Marital status, n(%)				0.006
Divorced	685 (13.33)	516 (12.77)	169 (15.43)	
Living with partner	186 (3.62)	135 (3.34)	51 (4.66)	
Married	2771 (53.94)	2211 (54.70)	560 (51.14)	
Never married	523 (10.18)	430 (10.64)	93 (8.49)	
Separated	198 (3.85)	155 (3.83)	43 (3.93)	
Widowed	774 (15.07)	595 (14.72)	179 (16.35)	
Education, n(%)				0.072
below high school	1860 (36.21)	1435 (35.50)	425 (38.81)	
high school	1163 (22.64)	913 (22.59)	250 (22.83)	
over high school	2114 (41.15)	1694 (41.91)	420 (38.36)	
Income, n(%)				<0.001
<20,000	1715 (33.39)	1302 (32.21)	413 (37.72)	
>=20,000	3422 (66.61)	2740 (67.79)	682 (62.28)	
Smoke, n(%)				0.972
No	2606 (50.73)	2050 (50.72)	556 (50.78)	
Yes	2531 (49.27)	1992 (49.28)	539 (49.22)	
Alcohol, n(%)				0.079
No	1651 (32.14)	1275 (31.54)	376 (34.34)	
Yes	3486 (67.86)	2767 (68.46)	719 (65.66)	
HBP, n(%)				0.003
No	3671 (71.46)	2928 (72.44)	743 (67.85)	
Yes	1466 (28.54)	1114 (27.56)	352 (32.15)	

The data represented the baseline demographic and clinical characteristics of the study population. Continuous variables are expressed as mean ± SD, and categorical variables as n (%). P-values were calculated using Student’s t-test for continuous variables and Chi-square test for categorical variables. Abbreviations: DR, diabetic retinopathy; SD, standard deviation; BMI, body mass index; HHR, HbA1c-to- HDL Ratio; SBP, systolic blood pressure; DBP, diastolic blood pressure; FBG, fasting blood glucose; HbA1c, hemoglobin A1c; HDL, high-density lipoprotein; LDL, low-density lipoprotein; TyG, triglyceride-glucose index; METS-IR, metabolic score for insulin resistance; NLR, neutrophil-to-lymphocyte ratio; MLR, monocyte-to-lymphocyte ratio; SII, systemic immune-inflammation index; SIRI, system inflammation response index; WBC, white blood cell.

### 3.2 Multivariate logistic regression model of blood urine routine, combined indicators and DR

To identify independent predictors of DR, we constructed a multivariable logistic regression model incorporating covariates that demonstrated significance in the univariate analysis (**Table 2**).

**Table 2:** Multivariate logistic regression model of blood and urine routine, combined indicators and diabetic retinopathy (only variables with P < 0.1 are presented).

Variables	β	P	OR (95%CI)
TyG	-0.01	0.042	0.99 (0.99 ~ 0.99)
TyG-BMI	0.00	0.061	1.00 (1.00 ~ 1.00)
Blood urea nitrogen	0.03	<0.001	1.03 (1.02 ~ 1.04)
Creatinine	0.12	0.027	1.13 (1.01 ~ 1.25)
Lactate dehydrogenase	0.01	<0.001	1.01 (1.01 ~ 1.01)
Total bilirubin	-0.28	0.036	0.76 (0.59 ~ 0.98)
Potassium	0.40	0.012	1.50 (1.26 ~ 1.77)
Sodium	-0.03	0.050	0.97 (0.94 ~ 1.00)
METS-IR	0.01	0.020	1.01 (1.00 ~ 1.02)
Insulin	0.01	0.040	1.01 (1.01 ~ 1.01)
PRO	0.01	<0.001	1.01 (1.01 ~ 1.01)
UCREA	-0.01	0.005	0.99 (0.99 ~ 0.99)
HbA1c	0.16	<0.001	1.18 (1.07 ~ 1.29)
DBP	-0.01	0.037	0.99 (0.99 ~ 0.99)
LHR	-2.29	0.058	0.10(0.01 ~ 1.41)
FBG	0.01	0.047	1.01 (1.00 ~ 1.05)

P values were derived from multivariate logistic regression analysis. TyG, Triglyceride-glucose index; TyG-BMI, Triglyceride-glucose-body mass index; METS-IR: Metabolic score for insulin resistance; PRO, Proteinuria; UCREA: Urine creatinine; HbA1c, Hemoglobin A1c; DBP, Diastolic blood pressure; LHR, LDL-to-HDL ratio; FBG, Fasting blood glucose; OR, Odds Ratio; CI, Confidence Interval.

Metabolic, Glycemic, and Lipid Profiles: Poor glycemic control and hyperinsulinemia emerged as primary risk factors. Specifically, elevated HbA1c (OR = 1.18, 95% CI: 1.07-1.29, P < 0.001) and fasting blood glucose (OR = 1.02, 95% CI: 1.00-1.05, P = 0.047) were independently associated with DR. This risk profile was compounded by insulin resistance, evidenced by increased serum insulin (OR = 1.01, 95% CI: 1.01-1.01, P = 0.040) and a higher METS-IR index (OR = 1.01, 95% CI: 1.00-1.02, P = 0.020), despite a marginal inverse association with the TyG index (OR = 0.99, P = 0.042). Furthermore, we evaluated the LHR as a composite lipid index. Although it demonstrated a broad inverse trend in this linear model, the association did not reach strict statistical significance (OR = 0.10, 95% CI: 0.01-1.41).

Renal and Urinary Indicators: Markers of latent renal impairment and altered urinary excretion were prominently linked to DR pathogenesis. Elevated BUN (OR = 1.03, 95% CI: 1.02-1.04, P < 0.001) and serum creatinine (OR = 1.13, 95% CI: 1.01-1.25, P = 0.027) strongly predicted the presence of DR. Concurrently, increased PRO conferred a higher risk (OR = 1.01, 95% CI: 1.01-1.01, P < 0.001), whereas higher UCREA exhibited a significant protective association (OR = 0.99, 95% CI: 0.99-0.99, P = 0.005).

Electrolytes, Enzymes, and Hemodynamics: Systemic electrolyte shifts and markers of cellular stress further delineated the DR phenotype. Elevated serum potassium (OR = 1.50, 95% CI: 1.26-1.77, P = 0.012) and LDH (OR = 1.01, 95% CI: 1.01-1.01, P < 0.001) significantly amplified DR risk. Conversely, independent protective associations were identified for higher total bilirubin (OR = 0.76, 95% CI: 0.59-0.98, P = 0.036), serum sodium (OR = 0.97, 95% CI: 0.94-1.00, P = 0.050), and d DBP (OR = 0.99, 95% CI: 0.99-0.99, P = 0.037).

Collectively, these conventional linear regressions confirm that DR onset reflects a multifactorial pathophysiological cascade spanning glycemic toxicity, renal impairment, and systemic oxidative stress.

### 3.3 Feature selection

To establish a robust and generalizable predictive framework, the complete dataset derived from the NHANES database was randomly partitioned into a training cohort and an internal testing cohort at a 7:3 ratio. Baseline demographic and clinical characteristics were strictly balanced, with no significant statistical differences observed between the two sets (all P > 0.05).

**Figure 2:**
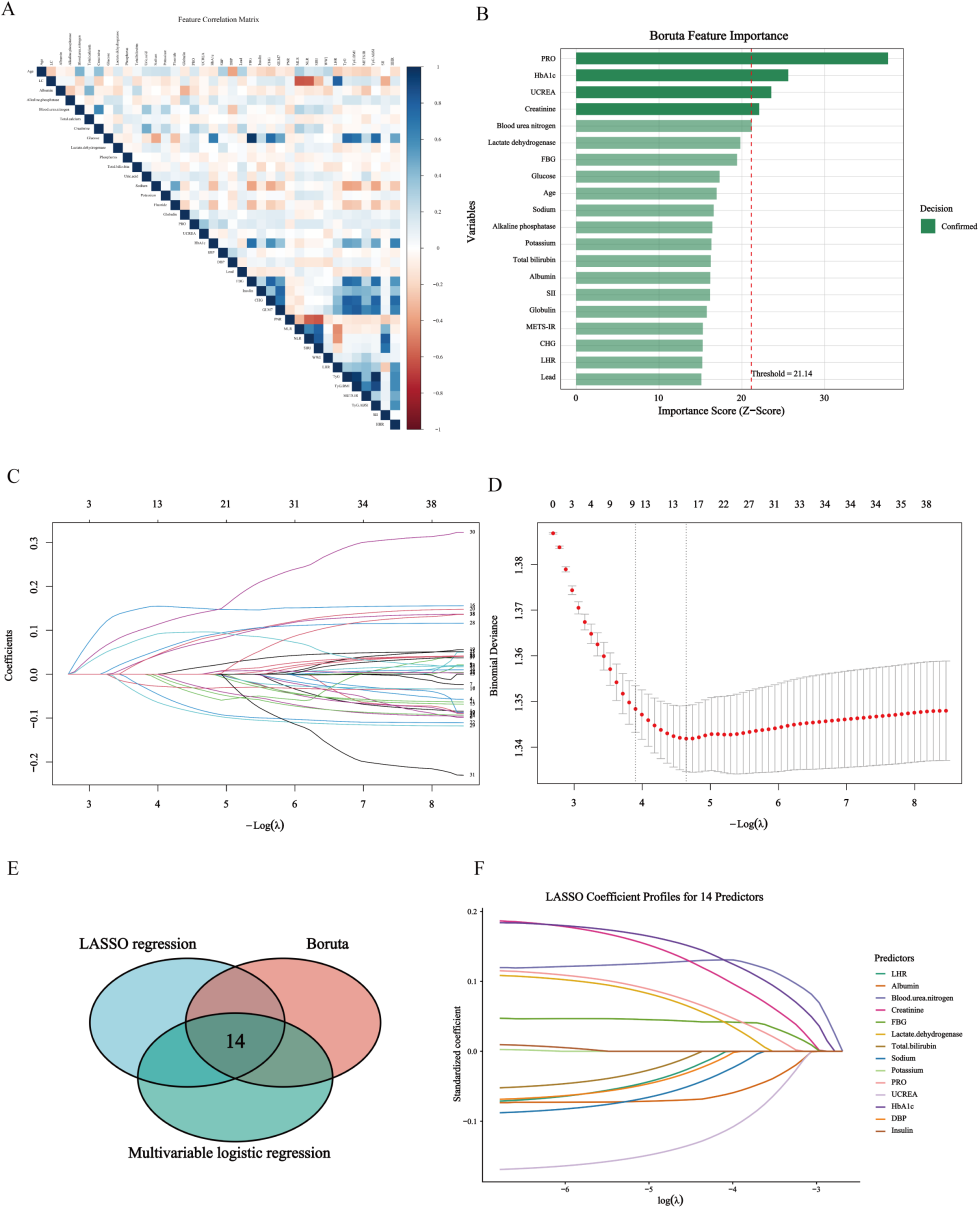
Multistage feature selection pipeline for identifying core predictors of diabetic retinopathy (DR). **(A)** Pairwise correlation matrix of baseline clinical variables. Color intensity represents the degree of correlation (Pearson’s or Spearman’s r), utilized to identify and mitigate multicollinearity among potential predictors. **(B)** Feature importance ranking derived from the Boruta algorithm. The random forest-based wrapper method classified variables as confirmed (important), tentative, or rejected based on their predictive relevance. **(C)** LASSO coefficient profiles of the candidate features. The vertical axis indicates the coefficient values, while the horizontal axis represents the logarithm of the penalty parameter (λ). **(D)** Ten-fold cross-validation curve for LASSO regression. The optimal λ value was selected at the minimum partial likelihood deviance to prevent overfitting. **(E)** Venn diagram illustrating the robust consensus of the feature selection process, showing the exact intersection of significant variables identified by both the LASSO regression and the multivariable logistic regression. **(F)** Confirmatory LASSO validation plot demonstrating the finalized 14 core predictors (LHR, Albumin, BUN, Creatinine, FBG, LDH, Total bilirubin, Sodium, Potassium, PRO, UCREA, HbA1c, DBP, and Insulin) utilized for subsequent machine learning model construction.

Prior to implementing advanced machine learning algorithms, a comprehensive pairwise correlation analysis was conducted to visualize variable interactions and mitigate the risk of multicollinearity (**Figure 2A**). Following the exclusion of highly redundant features, the Boruta algorithm, a robust, random forest-based wrapper method designed to capture all relevant features was deployed for preliminary screening (**Figure 2B**).

Candidate features confirmed by Boruta were subsequently subjected to Least Absolute Shrinkage and Selection Operator (LASSO) regression. To effectively penalize non-essential coefficients and prevent model overfitting, the optimal penalty parameter (λ) was rigorously determined through 10-fold cross-validation, minimizing the mean cross-validation error (**Figures 2C, D**). To ensure maximal statistical reliability, a Venn diagram was constructed to delineate the precise intersection of significant variables identified by both the LASSO regression and the Boruta algorithm models (**Figure 2E**).

This rigorous, multi-step intersecting pipeline successfully distilled the extensive feature pool down to a definitive set of 14 selected predictors: LHR, serum albumin, BUN, serum creatinine, FBG, LDH, serum total bilirubin, serum sodium, serum potassium, PRO, UCREA, HbA1c, DBP, and insulin. Before imputation, missingness across the 14 final predictors ranged from 7.34% to 10.77%. Most predictors showed similar missing rates between the DR and non-DR groups. However, PRO, UCREA and DBP exhibited significantly higher missingness in the DR group than in the non-DR group (P = 0.002 for PRO and UCREA; P = 0.0216 for DBP), whereas the remaining predictors showed no significant between-group differences. In the resampling-based stability analysis, the final selection frequencies of the 14 candidate core predictors were: BUN (100%), PRO (98%), HbA1c (97%), LDH (92%), Creatinine (90%), FBG (89%), UCREA (86%), Albumin (81%), Insulin (79%), Sodium (74%), DBP (71%), Potassium (69%), LHR (65%), and Bilirubin_Total (54%). Using a predefined threshold of 80%, eight predictors were identified as stable, including BUN, PRO, HbA1c, LDH, Creatinine, FBG, UCREA, and Albumin. To further confirm the validity of the selected feature set, a LASSO coefficient profile analysis was performed using all 14 finalized predictors (**Figure 2F**). In this plot, each colored curve corresponds to one predictor, and the y-axis reflects the standardized regression coefficient as a function of log(λ), where λ denotes the regularization parameter. As λ increased, the coefficients were progressively compressed toward zero, indicating increasing shrinkage and variable penalization. Predictors such as HbA1c, creatinine, BUN, LDH, and PRO retained relatively prominent coefficient magnitudes across a wide range of λ values, indicating greater robustness and stronger contribution to the penalized model. Overall, the orderly shrinkage patterns and sustained nonzero coefficients of several key predictors provide additional confirmation that the finalized 14-variable set possesses acceptable structural robustness and predictive validity.

### 3.4 Model development and performance comparison

Eight machine learning models were developed using fourteen routinely available biomarkers and showed varying discriminatory ability for identifying DR in adults with diabetes. In the training set, XGBoost achieved the highest apparent performance (AUC = 0.990), whereas LightGBM yielded an AUC of 0.946 (**Tables 3**, **4**). However, because training-set estimates are more susceptible to optimism and overfitting, final model selection was not based on training performance alone. Greater emphasis was placed on threshold-independent discrimination in the internal test set, with external validation used to further assess transportability and robustness.

**Table 3:** Predictive performance of machine learning models for diagnosing diabetic retinopathy in adults with diabetes within the training set.

Model	AUC	95%CI	Accuracy	Sensitivity	Specificity	F1
XGBoost	0.990	0.987-0.992	0.945	0.924	0.965	0.944
LightGBM	0.946	0.938-0.953	0.866	0.830	0.902	0.861
SVM	0.943	0.934-0.951	0.867	0.886	0.848	0.870
KNN	0.864	0.852-0.876	0.771	0.817	0.725	0.781
Random Forest	0.768	0.752-0.785	0.703	0.687	0.720	0.698
Decision Tree	0.728	0.710-0.745	0.701	0.705	0.695	0.701
Neural Network	0.712	0.693-0.730	0.660	0.650	0.671	0.657
Logistic Regression	0.647	0.627-0.666	0.603	0.511	0.695	0.562

The metrics (AUC, accuracy, sensitivity, specificity, F1-score) represent the diagnostic capability of each model within the internal testing cohort. AUC, area under the receiver operating characteristic curve.

**Table 4:** Predictive performance of machine learning models for diagnosing diabetic retinopathy in adults with diabetes within the internal test set.

Model	AUC	95%CI	Accuracy	Sensitivity	Specificity	F1
XGBoost	0.845	0.824-0.866	0.759	0.721	0.796	0.749
LightGBM	0.849	0.830-0.873	0.783	0.765	0.841	0.782
SVM	0.780	0.755-0.805	0.714	0.732	0.696	0.719
Random Forest	0.726	0.699-0.753	0.656	0.632	0.680	0.647
KNN	0.715	0.688-0.742	0.666	0.714	0.618	0.681
Decision Tree	0.666	0.637-0.695	0.641	0.648	0.633	0.644
Neural Network	0.664	0.635-0.693	0.613	0.586	0.639	0.602
Logistic Regression	0.645	0.615-0.674	0.585	0.479	0.691	0.536

The metrics (AUC, accuracy, sensitivity, specificity, F1-score) represent the diagnostic capability of each model within the internal testing cohort. AUC, area under the receiver operating characteristic curve.

**Figure 3:**
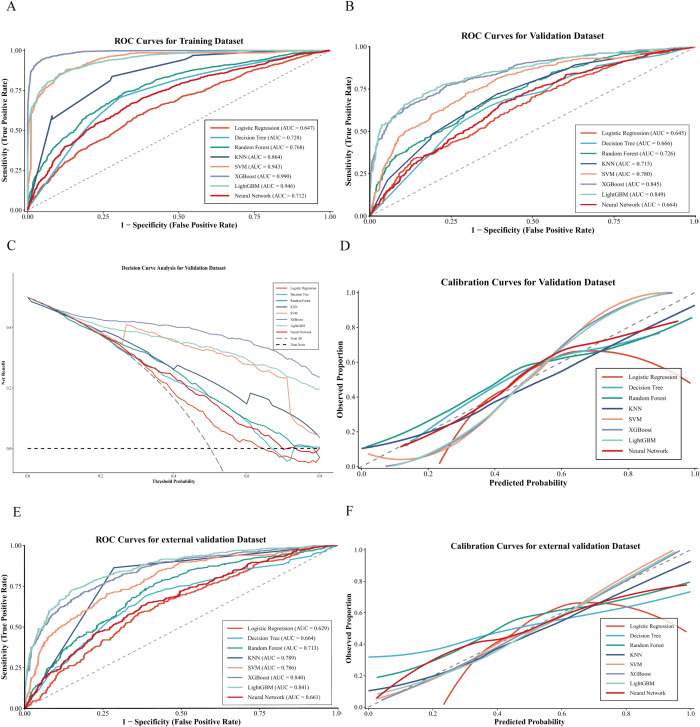
Performance evaluation and SHAP-based interpretability of machine learning models. **(A)** ROC curves illustrating the diagnostic performance of seven machine learning models in the training set. **(B)** ROC curves of the seven models in the testing set. **(C)** Calibration curves evaluating the agreement between predicted probabilities and observed frequencies in the testing set. **(D)** Decision curve analysis (DCA) assessing the clinical net benefit of the models in the testing set. **(E)** ROC curves illustrating the diagnostic performance of the seven machine learning models in the external validation set. **(F)** DCA evaluating the clinical net benefit of the seven models in the external validation set.

In the internal test set, LightGBM achieved the highest AUC (0.849), slightly exceeding XGBoost (0.845) (**Figures 3A, B**; **Tables 3**, **4**). In addition, the calibration curve and decision curve analysis (DCA) indicated that LightGBM had favorable calibration and potential clinical utility (**Figures 3C, D**). Because the primary objective of model comparison was discrimination across all possible thresholds, AUC was prespecified as the principal criterion for selecting the final model, whereas threshold-dependent metrics such as accuracy, sensitivity, specificity, and F1-score were considered supportive measures. On this basis, LightGBM was selected for subsequent interpretation and external validation.

### 3.5 External validation

External validation was performed using an independent clinical cohort from Nantong First People’s Hospital. In this cohort, LightGBM achieved the highest AUC (0.841), narrowly exceeding XGBoost (0.840), whereas KNN showed lower overall discrimination (AUC = 0.789) despite favorable threshold-dependent metrics at the selected cutoff (**Figures 3E, F**; **Table 5**). In the external validation cohort, the LightGBM model also demonstrated good calibration. The calibration-in-the-large was 0.009 (95% CI, -0.118 to 0.135), the calibration intercept was 0.035 (95% CI, -0.099 to 0.170), and the calibration slope was 1.151 (95% CI, 1.115 to 1.296). These results indicate minimal systematic over- or underestimation of risk overall, with only a mild tendency toward under-dispersion of predicted probabilities.

**Table 5:** Predictive performance of machine learning models for diagnosing diabetic retinopathy in adults with diabetes within the external validation cohort.

Model	AUC	95%CI	Accuracy	Sensitivity	Specificity	F1
XGBoost	0.840	0.815-0.866	0.755	0.747	0.769	0.751
LightGBM	0.841	0.809-0.862	0.758	0.740	0.769	0.756
KNN	0.789	0.762-0.815	0.789	0.863	0.715	0.803
SVM	0.786	0.756-0.816	0.699	0.731	0.667	0.708
Random Forest	0.713	0.679-0.747	0.644	0.580	0.708	0.620
Decision Tree	0.664	0.628-0.700	0.644	0.626	0.662	0.637
Neural Network	0.661	0.625-0.697	0.621	0.610	0.632	0.617
Logistic Regression	0.629	0.592-0.665	0.592	0.491	0.694	0.546

The metrics (AUC, accuracy, sensitivity, specificity, F1-score) represent the diagnostic capability of each model within the external validation cohort. AUC, area under the receiver operating characteristic curve.

It should be noted that KNN yielded higher sensitivity (0.863 vs. 0.740), accuracy (0.789 vs. 0.758), and F1-score (0.803 vs. 0.756) than LightGBM in the external validation cohort. However, because the primary model selection criterion in this study was AUC, which reflects threshold-independent discrimination across all possible operating points, LightGBM remained preferable in terms of overall discriminative ability. Moreover, LightGBM showed greater cross-dataset stability in AUC, decreasing from 0.946 in the training set to 0.849 in the internal test set and 0.841 in the external validation cohort. By comparison, XGBoost showed a larger decline from 0.990 to 0.845 and 0.840, respectively.

Although a modest attenuation in predictive performance was observed from internal testing to external validation, such variation is expected in independent validation across heterogeneous clinical populations. Taken together, these findings suggest that LightGBM provided the most favorable balance of threshold-independent discrimination, calibration, and cross-cohort stability. It was therefore retained as the final model for subsequent interpretation, web-based deployment, and individualized DR risk stratification.

### 3.6 SHAP-based model interpretation

To elucidate the inner workings of the optimal LightGBM model and identify the most influential risk biomarkers, SHAP (SHapley Additive exPlanations) analysis was conducted. This game-theoretic approach provides a transparent, model-agnostic framework to interpret complex, non-linear feature interactions, enabling a precise, quantitative assessment of each variable’s global and local contribution to the prediction outcomes.

Based on the mean absolute SHAP values, the top three features exerting the strongest magnitude of influence on increased DR risk were elevated LHR, PRO, and UCREA. Following closely, higher concentrations of HbA1c, sodium and total bilirubin also demonstrated substantial impact on the algorithmic decision-making process. Conversely, markers such as serum creatinine and BUN served as essential components of the predictive architecture but exhibited relatively modest global contributions compared to the primary glycemic and renal metrics.

**Figure 4:**
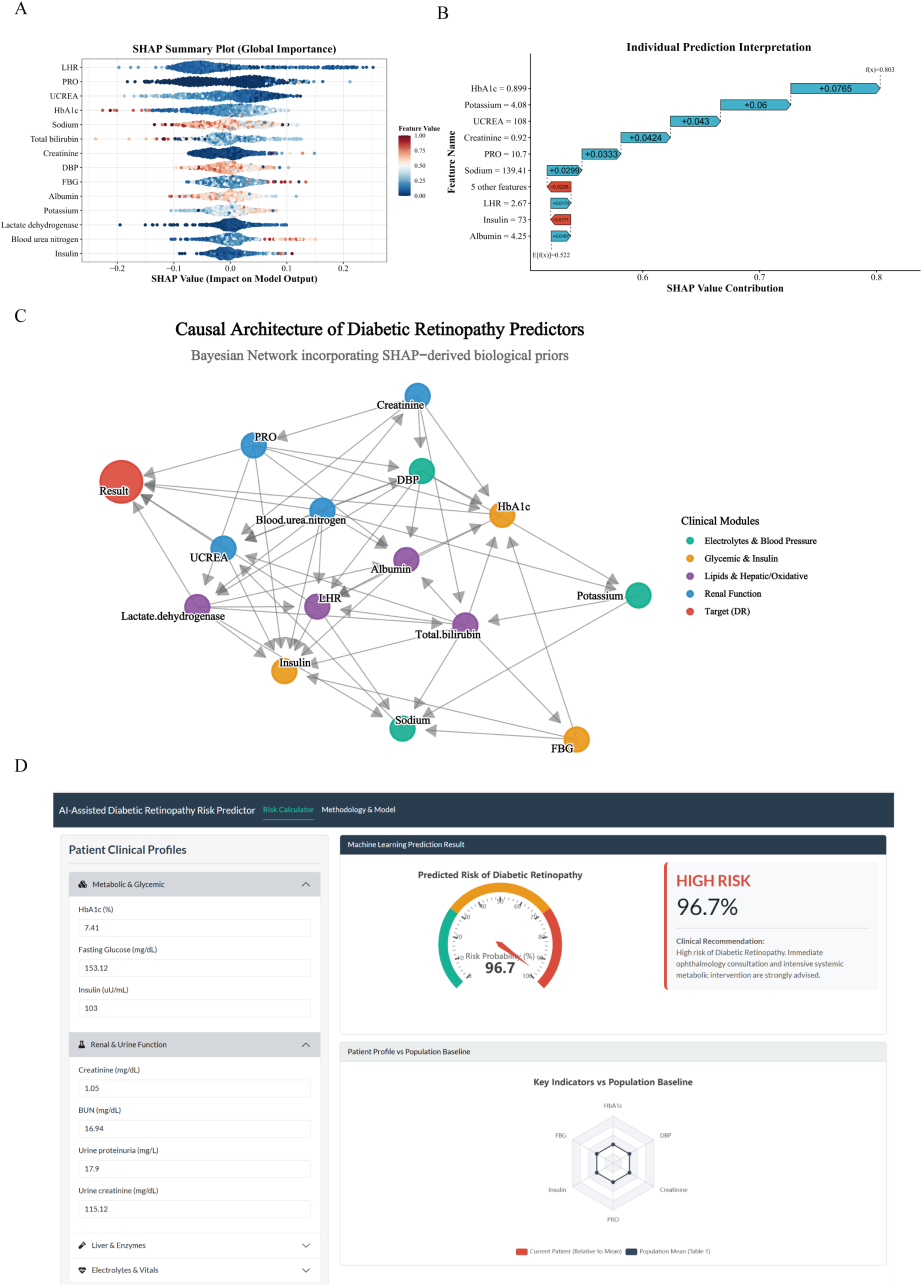
Performance validation in the external dataset and web-based model implementation. **(A)** SHAP summary plots for the optimal LightGBM model, comprising a beeswarm plot (illustrating feature density and impact direction) and a bar plot (ranking global feature importance). **(B)** SHAP waterfall plot demonstrating the contribution of individual features to a specific prediction case. **(C)** Bayesian Network Directed Acyclic Graph (DAG) mapping the dependency and hierarchical interdependencies among the 14 core systemic predictors of Diabetic Retinopathy (DR). **(D)** Interface of the web-based risk calculator based on the optimal LightGBM model, deployed via RStudio Server to facilitate personalized clinical risk assessment.

Beyond global feature ranking, local interpretability was achieved by extracting individual-level risk profiles. SHAP values for global interpretation were computed using the finalized LightGBM model on the testing cohort. Waterfall plots were generated to visualize patient-specific predictions, illustrating how the baseline values of each biomarker cumulatively pushed the model output toward or away from a DR diagnosis, including both the directionality and magnitude of contribution. The case shown in **Figure 4B** is presented as a representative illustrative example rather than a formal expert-adjudicated validation of individual-level explanations. (**Figures 4A, B**).

### 3.7 Directed dependency structure of DR predictors

To further decipher the mechanistic interplay among the 14 core predictors identified by the machine learning pipeline, a Bayesian Network (BN) Directed Acyclic Graph (DAG) was constructed (**Figure 4C**). Unlike SHAP values which rank parallel importance, the BN algorithm successfully delineated a probabilistic dependency structure. The network visually centralizes DR (Result) as the pathogenetic sink. Notably, the graph maps a clear glycemic cascade where FBG acts as an upstream driver of HbA1c, which subsequently imposes a direct effect on DR. More importantly, markers of renal impairment—specifically PRO, BUN, and UCREA—emerged as dominant direct predecessors to DR, being heavily driven by upstream hemodynamic (DBP) and metabolic factors.

### 3.8 Clinical implementation via web application

To bridge the gap between complex algorithmic development and practical healthcare delivery, the optimized LightGBM model was successfully translated into a server-based, interactive web application (currently undergoing internal beta testing) designed for real-time clinical risk stratification (**Figure 4D**). A paramount advantage of this deployment is its exclusive reliance on highly accessible, low-cost clinical parameters—specifically, routine physical examination metrics alongside standard comprehensive metabolic and urinalysis panels.

Within the intuitive user interface, clinicians can swiftly input the patient’s values for the 14 finalized core predictors. To ensure data integrity and prevent erroneous algorithmic extrapolations, the system’s sidebar explicitly defines physiological reference ranges for each continuous variable. Any input values deviating beyond these predefined biological limits are automatically flagged, truncated, or processed as missing data, thereby maintaining the robust boundaries of the model’s training space.

Upon data entry, the tool instantly generates a comprehensive, personalized clinical report comprising three critical dimensions. Primarily, it yields a real-time risk quantification, outputting the exact percentage probability of DR presence for the specific patient. Simultaneously, the system enhances algorithmic transparency by rendering an interactive SHAP force plot. This dynamic visual interpretation demystifies the predictive “black box,” elegantly illustrating how specific patient baseline values act as physiological forces, pushing the predictive needle toward either a high-risk or low-risk outcome. Currently undergoing internal beta testing, this web-based prototype represents an evidence-based clinical decision support system (CDSS) that has the potential to empower frontline healthcare providers with a highly interpretable and accessible tool for the early identification and proactive management of diabetic retinopathy, pending future public deployment.

From a deployment perspective, the final LightGBM model uses a limited number of routinely available predictors and is computationally lightweight. As a result, once trained, the model is suitable for near real-time risk estimation in a web-based application. In addition, SHAP-based local explanation is feasible at the individual patient level given the modest feature dimension and tree-based model structure. Nevertheless, because this study focused on model development and validation rather than production-level implementation benchmarking, formal evaluation of training time, inference latency, and explanation generation across hardware environments remains an important direction for future work.

## 4 Discussion

This dual-center study successfully developed, externally validated, and deployed a highly interpretable machine learning (ML) framework for the risk stratification of DR. By shifting the diagnostic paradigm away from specialized imaging toward routine, highly accessible laboratory biomarkers, we address a critical bottleneck in global DR screening. Among the evaluated algorithms, the LightGBM model, refined through a Boruta-LASSO feature selection pipeline, demonstrated superior discriminative capacity, achieving an AUC of 0.849 in the internal testing set. Crucially, the model yielded a highly robust AUC of 0.841 in the independent external validation cohort. This minimal performance attenuation (ΔAUC = 0.008) across distinct hospital populations strongly underscores the algorithm’s exceptional generalizability and its resilience against clinical data heterogeneity. By integrating SHAP analysis and deploying the model as a web-based clinical decision support system (CDSS), this study effectively bridges the gap between complex mathematical computations and pragmatic clinical utility.

Currently, the vanguard of artificial intelligence in ophthalmology is dominated by deep learning models trained on fundus photographs or OCT (30), and recent comparative studies have further shown the potential of deep learning for enhanced prediction of diabetic retinopathy in diabetes complications datasets (31). This should also be considered when interpreting the scope of our model benchmarking, as deep neural network architectures were not included in the original comparison framework, which focused on structured laboratory and clinical variables rather than image-based inputs. While these imaging-based algorithms achieve remarkable diagnostic accuracy, their real-world implementation in primary care or resource-constrained settings is frequently hindered by prohibitive equipment costs, a shortage of specialized technicians, and the requirement for pupillary dilation. In contrast, our approach circumvents these systemic barriers by relying exclusively on 14 universally available peripheral blood and urinalysis parameters. This data-driven, non-invasive strategy serves as a cost-effective “gatekeeper,” facilitating seamless integration into existing electronic health record (EHR) systems to enable automated, real-time risk surveillance prior to specialist referral.

Beyond robust algorithmic performance, the feature importance derived from our SHAP analysis provides profound mechanistic insights into the multifactorial pathophysiology of DR, corroborating the hypothesis that retinal microangiopathy is a localized manifestation of profound systemic dysregulation (32). Notably, the model identified elevated PRO, UCREA, BUN, and serum creatinine as pivotal drivers of DR risk. This strongly aligns with the concept of “diabetic renoretinal syndrome” or “kidney-eye crosstalk” (33). Given that the retinal and glomerular microvasculature share analogous anatomical architectures and developmental pathways, pathophysiological cascades—such as endothelial dysfunction, basement membrane thickening, and pericyte loss—often manifest concurrently in both organs under chronic hyperglycemic stress.

Furthermore, our model captures the complex interplay of non-traditional metabolic and inflammatory markers. The inclusion of the LHR highlights the critical role of atherogenic dyslipidemia and systemic lipotoxicity in compromising the blood-retinal barrier. Interestingly, total bilirubin and LDH emerged as significant independent predictors. LDH is a well-established surrogate for cellular hypoxia and progressive tissue damage. Conversely, mild elevations in total bilirubin have been paradoxically associated with endogenous antioxidant effects, offering a protective mechanism against diabetic microvascular complications by neutralizing ROS (34). The autonomous identification of these specific prognostic features by the LightGBM algorithm mirrors the unifying theory of diabetic complications, wherein hyperglycemia-induced superoxide overproduction acts as the central engine of vascular tissue damage (35).

A fundamental barrier to the clinical adoption of advanced machine learning models is the black box dilemma, defined as the inherent opacity regarding how algorithms compute patient specific predictions (36). Our study addressed this barrier by employing SHAP values to generate both global feature rankings and individual level waterfall plots. However, while SHAP elegantly quantifies the magnitude of feature contributions, it fundamentally relies on correlative associations rather than directed biological causality. To transcend this methodological limitation, we introduced a Bayesian Network Directed Acyclic Graph to map the hierarchical pathogenetic architecture of the core predictors. As highlighted by recent methodological advancements in medical artificial intelligence (37), integrating inference with predictive modeling is crucial to prevent the learning of spurious correlations and to ensure that algorithms align with physiological reality. By utilizing SHAP derived feature importance as biological priors, our Bayesian Network successfully overcame collinearity masking among highly correlated physiological indices. The resulting topology identified chronic glycemic toxicity and latent renal impairment as probabilistically dominant upstream nodes converging on the DR outcome. This dual layer framework transforms abstract probabilities into actionable and biologically plausible clinical intelligence, empowering primary care physicians to comprehend whether a patient’s elevated risk is driven primarily by poor glycemic control, latent renal endothelial damage, or lipid disturbances.

From a clinical implementation perspective, the web-based model is intended as a risk-stratification and referral-triage tool rather than a replacement for fundus examination. It may be particularly useful in endocrinology clinics, primary care settings, and community-based diabetes management programs, where ophthalmic resources are limited or retinal imaging is not immediately available. In such settings, the tool can support prioritization of patients for ophthalmic referral and timely fundus examination. In this study, we propose a predicted probability threshold of 0.20 as a practical cutoff for action. Patients with predicted probabilities at or above this level may be considered for prompt referral for retinal evaluation, whereas those below this threshold should continue routine diabetes care and standard retinal screening schedules rather than being regarded as not requiring eye examination.

Despite its rigorous design including multistage feature selection and independent external validation, this study possesses certain limitations. First, the cross sectional and retrospective nature of the training and validation cohorts inherently restricts the definitive confirmation of longitudinal causality. Although our Bayesian Network provides robust mathematical evidence for a directed pathogenetic cascade, future investigations employing Mendelian randomization or large scale prospective cohorts remain necessary to definitively substantiate these probabilistic dependency structure in human physiology (38). Second, although we comprehensively adjusted for multiple parameters, potential unmeasured confounders could not be fully accounted for. These unmeasured factors include exact diabetes duration, dietary habits, and the specific use of novel antidiabetic agents (such as GLP-1 receptor agonists or SGLT2 inhibitors) that are known to confer pleiotropic microvascular protection. SGLT2 inhibitors are known to improve renal outcomes, including reducing albuminuria/proteinuria and slowing kidney function decline, whereas GLP-1 receptor agonists may influence vascular and metabolic pathways relevant to DR. In addition, rapid glycemic improvement after initiation of some glucose-lowering therapies has been associated with early worsening of retinopathy in certain settings (39–41). Therefore, the observed roles of renal-related markers in our model may partly reflect unmeasured treatment effects rather than solely underlying disease mechanisms. These associations should thus be interpreted with caution, and future studies with detailed medication records are warranted. Third, while external validation confirmed the structural resilience of the model, evaluating the real world clinical impact of the prototype on physician decision making and patient outcomes via prospective randomized controlled trials remains a requisite step prior to widespread rollout.

Fourth, although the use of NHANES as the derivation cohort and a Chinese hospital cohort for external validation provided an opportunity to preliminarily assess cross-population transportability, the potential influence of racial and ethnic heterogeneity on model performance should be acknowledged. The NHANES dataset represents a U.S.-based multi-ethnic population, whereas the external validation cohort consisted exclusively of Chinese patients from a single tertiary center in Nantong. Prior studies have shown that diabetes phenotypes, adiposity patterns, insulin secretion and resistance, metabolic profiles, and susceptibility to diabetic microvascular complications may differ substantially across racial and ethnic groups (42). Such differences may affect both the baseline prevalence of DR and the predictive relationships between routine laboratory biomarkers and retinopathy risk. This concern may be particularly relevant for renal-related markers, since the burden and progression patterns of diabetic kidney disease and albuminuria also vary across populations (33). Therefore, despite the encouraging external validation performance observed in our study, the model’s generalizability to other racial, ethnic, and geographic populations should be interpreted with caution. Future studies should prioritize broader multicenter validation in ethnically diverse populations, subgroup performance analyses by race/ethnicity, and, where necessary, recalibration or model updating to improve robustness, fairness, and clinical applicability across settings (36).

Furthermore, diabetes subtype could not be reliably distinguished within NHANES. Although the study population consisted of adults and therefore predominantly represented type 2 diabetes, a small proportion of type 1 diabetes cases may have been included. Another limitation of this study is that diabetes duration, although an established risk factor for DR, was not retained in the final model because many patients in the external validation cohort could not provide a reliable duration history. Future studies with more complete longitudinal records should further evaluate its incremental predictive value. Importantly, while our Bayesian network analysis visually illustrates a significant probabilistic dependency between renal function markers (such as BUN, creatinine, urine creatinine, and proteinuria) and DR risk—supporting the hypothesized “kidney-eye crosstalk” in diabetic microvascular complications—these links should be interpreted with caution. In diabetic individuals with coexisting non-diabetic kidney disease, abnormalities in renal biomarkers may not specifically reflect diabetic microvascular renal injury, but may instead arise from alternative nephrologic etiologies. Therefore, the renal markers incorporated into our model should be understood primarily as predictive clinical signals associated with DR risk rather than definitive etiologic indicators of diabetic nephropathy. Likewise, the directed dependencies inferred by the Bayesian network represent population-level probabilistic relationships within the observational dataset, rather than proof of a unidirectional mechanism in every patient. Future studies with more detailed renal phenotyping, exclusion of known non-diabetic kidney disease, or stratified sensitivity analyses will be needed to further clarify the specificity of the observed kidney-eye interaction.

## 5 Conclusions

In conclusion, we established a highly robust and interpretable machine learning framework for diabetic retinopathy risk prediction utilizing fourteen routine clinical biomarkers. By successfully marrying the advanced predictive power of LightGBM with SHAP explainability and Bayesian network analysis of probabilistic dependencies, this study provides a scalable and transparent screening aid. This dual-layer analytical approach accurately stratifies high-risk patients and visually illustrates the probabilistic dependencies consistent with the kidney-eye crosstalk hypothesis. Ultimately, this tool holds immense promise for democratizing early diabetic retinopathy detection, optimizing healthcare resource allocation, and advancing personalized disease management strategies in primary care environments.

## Data Availability

The raw data supporting the conclusions of this article will be made available by the authors, without undue reservation.
